# Vertical sleeve gastrectomy normalizes circulating glucocorticoid levels and lowers glucocorticoid action tissue-selectively in mice

**DOI:** 10.3389/fendo.2022.1020576

**Published:** 2022-09-29

**Authors:** Elina Akalestou, Livia Lopez-Noriega, Ioannis Christakis, Ming Hu, Alexander D. Miras, Isabelle Leclerc, Guy A. Rutter

**Affiliations:** ^1^ Section of Cell Biology and Functional Genomics, Department of Metabolism, Digestion and Reproduction, Imperial College London, London, United Kingdom; ^2^ Endocrine and General Surgery, Nottingham University Hospitals NHS Trust, Nottingham, United Kingdom; ^3^ Section of Investigative Medicine, Department of Metabolism, Digestion and Reproduction, Imperial College London, London, United Kingdom; ^4^ Centre de Recherches du CHUM, University of Montreal, Montreal, QC, Canada; ^5^ Lee Kong Chian School of Medicine, Nanyang Technological University, Singapore, Singapore

**Keywords:** bariatric surgery, diabetes, adrenal glands, glucocorticoids, 11β-HSD1

## Abstract

**Objectives:**

Glucocorticoids produced by the adrenal cortex are essential for the maintenance of metabolic homeostasis. Glucocorticoid activation is catalysed by 11β-hydroxysteroid dehydrogenase 1 (11β-HSD1). Excess glucocorticoids are associated with insulin resistance and hyperglycaemia. A small number of studies have demonstrated effects on glucocorticoid metabolism of bariatric surgery, a group of gastrointestinal procedures known to improve insulin sensitivity and secretion, which were assumed to result from weight loss. In this study, we hypothesize that a reduction in glucocorticoid action following bariatric surgery contributes to the widely observed euglycemic effects of the treatment.

**Methods:**

Glucose and insulin tolerance tests were performed at ten weeks post operatively and circulating corticosterone was measured. Liver and adipose tissues were harvested from fed mice and 11β-HSD1 levels were measured by quantitative RT-PCR or Western (immuno-) blotting, respectively. 11β-HSD1 null mice (*Hsd11b1* ^-/-^) were generated using CRISPR/Cas9 genome editing. Wild type and littermate *Hsd11b1* ^-/-^ mice underwent Vertical Sleeve Gastrectomy (VSG) or sham surgery.

**Results:**

Under the conditions used, no differences in weight loss were observed between VSG treated and sham operated mice. However, both lean and obese WT VSG mice displayed significantly improved glucose clearance and insulin sensitivity. Remarkably, VSG restored physiological corticosterone production in HFD mice and reduced 11β-HSD1 expression in liver and adipose tissue post-surgery. Elimination of the 11β-HSD1/*Hsd11b1* gene by CRISPR/Cas9 mimicked the effects of VSG on body weight and tolerance to 1g/kg glucose challenge. However, at higher glucose loads, the euglycemic effect of VSG was superior to *Hsd11b1* elimination.

**Conclusions:**

Bariatric surgery improves insulin sensitivity and reduces glucocorticoid activation at the tissular level, under physiological and pathophysiological (obesity) conditions, irrespective of weight loss. These findings point towards a physiologically relevant gut-glucocorticoid axis, and suggest that lowered glucocorticoid exposure may represent an additional contribution to the health benefits of bariatric surgery.

## Introduction

Type 2 Diabetes (T2D) is a heterogeneous disease which most commonly presents as impaired insulin secretion or low insulin sensitivity (1). Despite an abundance of pharmacological, nutritional, exercise and behavioural interventions, approaches to treating the disease still focus on disease *management*, rather than *remission* (2, 3). Bariatric surgery, originally conceived as weight loss-aiding gastrointestinal surgery (4, 5), has been shown to cause long-term remission of T2D in many patients (6, 7). The effects of surgery go beyond reductions in adipose tissue mass and weight loss. Numerous studies (8, 9) have attempted to determine the exact pathways involved in order to replicate the beneficial metabolic effects of surgery in a less invasive way. These changes include substantial improvements in insulin sensitivity and hepatic gluconeogenesis alongside improvements in liver, cardiovascular, pancreatic islet and kidney function (10–12), pointing to a potential trans-organ communication axis. Importantly, the potential mechanisms investigated include possible increase in the secretion or action of multiple hormones in addition to insulin, such as the gut-derived incretin Glucagon-like Peptide 1 (GLP-1) (13). One possibility that remains largely unexplored, however, is the existence of a ‘gut-glucocorticoid’ axis, and specifically an altered role for glucocorticoids in regulating metabolism after bariatric surgery.

Glucocorticoids are produced by the adrenal cortex primarily under control of the hypothalamic-pituitary-adrenal (HPA) axis, but their secretion is also regulated by glucose regulating hormones such as insulin (14), glucagon (15) and GLP-1 (16). Glucocorticoids are activated at the tissue level by 11β-hydroxysteroid dehydrogenase 1 (11β-HSD1), which is present in most tissues (with the exception of pancreatic β-cells where this gene is “disallowed”) (17) and acts predominantly as an NADPH-dependent reductase to regenerate the active glucocorticoid receptor (GR) ligand cortisol (or corticosterone, in rodents) from inactive cortisone (18).

Although glucocorticoids are important for the maintenance of lipid homeostasis, excess glucocorticoids can result in an increase in the circulating free fatty acids and lipid accumulation in skeletal muscle and liver, both of which are associated with insulin resistance (19). Glucocorticoid metabolism is dysregulated in human obesity, where unbalanced plasma cortisol levels and adipose tissue 11β-HSD1 activity are observed (20, 21). Cortisol activation relies heavily on 11β-HSD1 and it has been suggested that the inhibition of this enzyme may provide a target for T2D and obesity treatments, acting to improve insulin sensitivity (22, 23).

Several observations have linked bariatric surgery to glucocorticoid metabolism, particularly in the context of tissular regulation of cortisol in obesity and post-operative weight loss (24, 25). Nonetheless, one study (26) showed that patients with obesity at one year post bariatric surgery (biliopancreatic diversion with duodenal switch) displayed lower adipose tissue 11β-HSD1 activity, when compared to normal weight unoperated controls. This finding points to an effect that could be caused by weight loss-independent mechanisms and may include the gastrointestinal tract manipulation itself as part of a gut-glucocorticoid axis. One potential mechanism could be the effect of GLP-1 on glucocorticoid regulation (27–29).

In the present study we tested the hypothesis that a reduction in glucocorticoid function following bariatric surgery contributes to the widely observed euglycemic effect. Specifically, we investigated the effect of Vertical Sleeve Gastrectomy (VSG) (30), on adrenal function under normal and obesity conditions, using lean and HFD mice, respectively.

## Materials and methods

### Animals

All animal procedures undertaken were approved by the British Home Office under the UK Animal (Scientific Procedures) Act 1986 (Project License PA03F7F0F to I.L.) with approval from the local ethical committee (Animal Welfare and Ethics Review Board, AWERB), at the Central Biological Services (CBS) unit at the Hammersmith Campus of Imperial College London. Purchased adult male (8 weeks old) C57BL/6J mice (Envigo, Huntingdon U.K.) were maintained and group-housed under controlled temperature (21-23°C) and light (12:12 hr light-dark schedule, lights on at 0700). The animals were fed either PMI Nutrition International Certified Rodent Chow No. 5CR4 (Research Diet, New Brunswick, NJ) or 58 kcal% Fat and Sucrose diet (D12331, Research Diet, New Brunswick, NJ) for twelve weeks and ad libitum. All mice were divided in two groups, VSG and control sham. A total of 30 animals were operated in lean state and HFD state. Liver and adipose tissue biopsies were removed from all mice at twelve weeks following sham or VSG surgery in the fed state, and were either snap frozen in -80°C, fixed in formalin, or both. Sample size was calculated according to predicted glycemia change between VSG and sham groups, using the results of previous study (12), as well as a max of 30% mortality prediction. Animals within cages were randomised according to their pre-operative body weight to ensure similar average between VSG and sham groups. EA conducted all experiments and was aware of treatment allocations.

During the Semaglutide study, lean adult male (8 weeks old) C57BL/6 mice were treated with either a single subcutaneous (SC) injection of Semaglutide (Novo Nordisk UK) at 5nmol/kg (n=5) or saline (n=5), for 7 and 30 days. Body weight was measured daily and all animals were euthanized and tissues were harvested on day 7 and day 30.

### Generation of 11β-HSD1 (Hsd11b1) null mice

Two gRNAs flanking exon1 region of *Hsd11b1* were designed using the software CRISPOR (http://crispor.tefor.net/). Both gRNAs were synthesized, mixed with Cas9 protein and microinjected into 30-50 mouse zygotes at the Medical Research Council transgenic facility in Imperial College London. F0 compound homozygous were crossed with wild type. F1 heterozygous mice were sequenced to determine whether exon1 is deleted. Heterozygous mice positive for exon1 deletion were then crossed to generate wild-type, heterozygous and homozygous littermates. Genotyping PCR reaction was carried out using the primer set listed in **Table 1**. PCR products were then column-purified (PCR purification, Qiagen) and sent for Sanger sequencing (Genewiz, Germany). A group of 14 animals underwent VSG and sham surgery. A group of littermate Wild Type mice (n=5) was also sham operated and function as an additional control.

**Table 1:** Primers Used for the Quantitative Detection of Hsd11b1, Nr3c1, Pck1, G6pc2 normalised to β−actin.

Analysed Transcript	Sequence of FW primer	Sequence of RV primer
Hsd11b1 ex1	TGCCTGGGAGGTTGTAGAAAG	CCCTGGAGCATTTCTGGTCTG
Hsd11b1 ex2	GTGATTGTCACTGGGGCCAGCAAAG	CAAATGTCATGTCTTCCATAGTGC
Hsd11b1 ex3	GACGACATCCACTCTGTGCGAAG	CTGTGTCTATGAGGCCAAGGACAC
Hsd11b1 GT	GCTATCTGGATGAGCCCTGTGTCTGG	ACAGTCATGAGCCTGGCCATCTGG
Nr3c1 (GR)	GCAGTGGAAGGACAGCACAA	GAGACTCCTGCAGTGGCTTG
Pck1	CCAAAAGGAAGAAAGGTGGCA	GTGGATATACTCCGGCTGGC
G6pc1	CTCCCAGGACTGGTTCATCC	TGACGTTCAAACACCGGAATC
Gck	TGGTGGATGAGAGCTCAGTGAA	CATGTACTTTCCGCCAATGATC
β -actin	CACTGTCGAGTCGCGTCC	TCATCCATGGCGAACTGGTG

### Vertical sleeve gastrectomy

Following 12 weeks of High Fat Diet animals underwent Vertical Sleeve Gastrectomy or sham surgery, as previously described (11, 12). Three days before bariatric or sham surgery, animals were exposed to liquid diet (20% dextrose) and remained on this diet for up to four days post operatively. Following this, mice were returned to high fat/high sucrose diet until euthanasia and tissues harvested ten weeks post bariatric surgery. Anaesthesia was induced and maintained with isoflurane (1.5-2%). A laparotomy incision was made, and the stomach was isolated outside the abdominal cavity. A simple continuous pattern of suture extending through the gastric wall and along both gastric walls was placed to ensure the main blood vessels were contained. Approximately 60% of the stomach was removed, leaving a tubular remnant. The edges of the stomach were inverted and closed by placing two serosae only sutures, using Lembert pattern (31). The initial full thickness suture was subsequently removed. Vicryl absorbable sutures (8.0) were used. Sham surgeries were performed by isolating the stomach and performing a 1 mm gastrotomy on the gastric wall of the fundus. All animals received a three-day course of analgesics Carprofen (Bayer, UK) and a five-day course of SC antibiotic injections (Enrofloxacin 10mg/kg). A survival of 80-90% was achieved.

### Glucose tolerance tests

Mice were fasted either overnight (total 16 h) or for 8hrs and given free access to water. Glucose (1 or 3 g/kg body weight) was administered *via* intraperitoneal injection or oral gavage. Blood was sampled from the tail vein at 0, 5, 15, 30, 60 and 90 min. after glucose administration. Blood glucose was measured with an automatic glucometer (Accuchek; Roche, Burgess Hill, UK). Any animals that did not appear well (mobile, grooming, fighting) were excluded from the experiment.

### Insulin tolerance tests

Mice were fasted for 8 h and given free access to water. At 1500, human insulin (Actrapid, Novo Nordisk) (0.8 or 1.5U/kg body weight) was administered *via* intraperitoneal injection. Blood was sampled from the tail vein at 0, 15, 30, 60 and 90 min after insulin administration. Blood glucose was measured with an automatic glucometer (Accuchek; Roche, Burgess Hill, UK). Any animals that did not appear well (hypoglycaemia <1.5 mmol/L, mobile, grooming) were excluded from the experiment.

### Plasma corticosterone measurement

To quantify circulating corticosterone levels, 50μl of blood was collected from the tail vein into heparin-coated tubes (Sarstedt, Beaumont Leys, UK) at 0800 and 1900. Plasma was separated by sedimentation at 10,000 ***g*** for 10 min. (4°C). Plasma corticosterone levels were measured in 10μl aliquots ELISA kits from Crystal Chem (USA).

### Plasma insulin and GLP-1 measurement

To quantify circulating insulin and GLP-1(1-37) levels, 100μl of blood was collected from the tail vein into heparin-coated tubes (Sarstedt, Beaumont Leys, UK). Plasma was separated by sedimentation at 10,000 ***g*** for 10 min. (4°C). Plasma insulin levels were measured in 5μl aliquots and GLP-1(1-37) levels were measured in 10μl aliquots by ELISA kits from Crystal Chem (USA).

### Western (immuno-) blotting

Liver and adipose tissue were lysed in ice-cold RIPA buffer containing a protease inhibitor mixture (Roche) and phosphatase inhibitors (Sigma-Aldrich). Lysates were denatured for 5 min at 95°C in Laemmli buffer, resolved by 10% SDS-PAGE, and transferred to polyvinylidene difluoride membranes before immunoblotting. The following antibodies were used: anti-rabbit 11β-HSD1 (Abcam, USA) (1:200) and anti-mouse GAPDH (Sigma-Aldrich) (1:1000). Intensities were quantified using ImageJ.

### RNA extraction, cDNA synthesis and quantitative polymerase chain reaction

Tissues were harvested and snap-frozen in liquid nitrogen. RNA was purified using PureLink RNA kit (Thermo Fisher Scientific, UK), according to manufacturers instructions. The purified RNA was dissolved in RNase and DNase free distilled water (Thermo Fisher Scientific, UK) and was immediately stored at −80°C until further analysis. Complementary DNA was synthesized from total RNA with High-Capacity cDNA Reverse Transcription Kit (Thermo Fisher Scientific, UK), according to the protocol recommended by the manufacturer. Quantitative Reverse Transcription PCR (qRT-PCR) analysis was used to quantify the expression level of *Hsd11b1, Pck1, G6pc* and *Nr3c1* (GR) mRNA in liver and adipose tissue. Primers, which crossed a splice junction, were designed using Primer Express (Invitrogen, UK; **Table 1**). The expression levels were measured by Q-PCR, using Fast SYBR Green Master Mix (Invitrogen) and a 7500 Fast Real-Time PCR System (Applied Biosystems, UK). Data from were normalized against β-actin levels. The analytical method used was 2(−Delta Delta Ct) (2–ΔΔCt).

### Statistical analysis

Statistical Analysis - Data were analysed using GraphPad PRISM 9.0 software. Significance was tested using non-parametric, unpaired (or paired in the case of corticosterone and GLP-1) Student’s two-tailed t-tests, or 2-way ANOVA (adjusted for multiple comparisons) as indicated. P<0.05 was considered significant and errors signify ± SEM.

## Results

### Vertical Sleeve Gastrectomy improves glucose tolerance and restores insulin sensitivity

**Figure 1:**
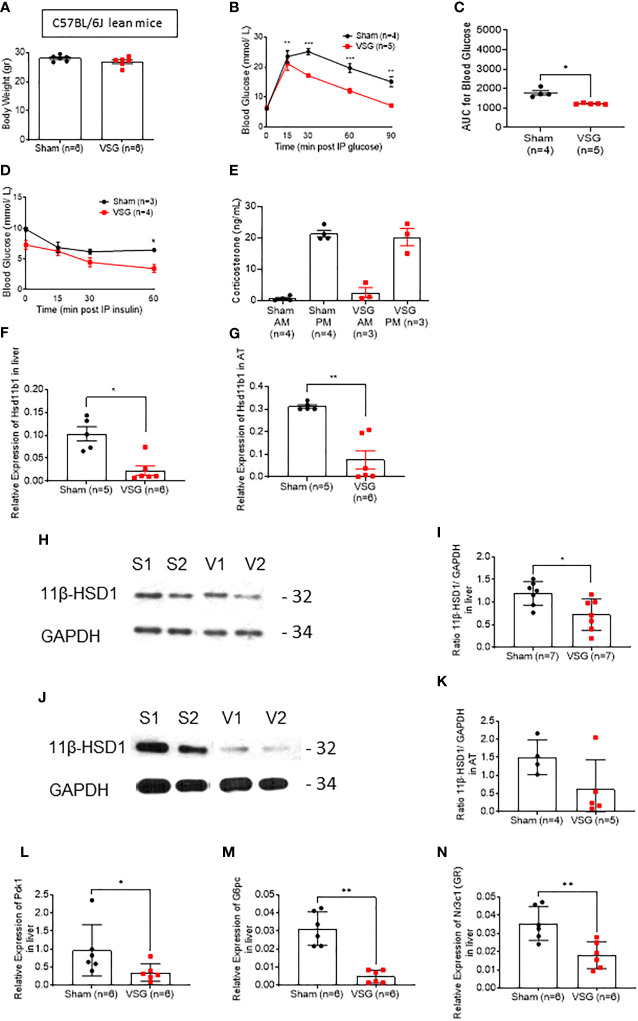
VSG improves glucose and insulin tolerance in lean mice. **(A)** Body weight at ten weeks following VSG (n = 6) or sham surgery (n = 6) in lean animals **(B)** IP glucose tolerance test in lean mice (3 g/kg) overnight fasting 4 weeks after surgery *P < 0.05, **P < 0.01, ***P < 0.001 Sham vs. VSG following 2-way ANOVA **(C)** Area under the Curve of **(B)**. *P < 0.05 Sham vs. VSG by two-sided unpaired Student’s t–test **(D)** Insulin tolerance test in lean mice (0.8 IU/kg) 6hr fasting, 4 weeks after surgery **(E)** Corticosterone measurement following blood collection at 0800 and 1900 within the same day, 4 weeks after surgery **(F)** Quantitative PCR levels of *Hsd11b1* gene expression in liver *P < 0.05 Sham vs. VSG by two-sided unpaired Student’s t–test **(G)** Quantitative PCR levels of *Hsd11b1* gene expression in subcutaneous adipose tissue **P < 0.01 Sham vs. VSG by two-sided unpaired Student’s t–test **(H)** Western Blot of liver lysate from sham and VSG mice for 11β-HSD1 (32kDa) and GAPDH (34kDa) **(I)** Average intensity measurement from Western Blot immunoblotting quantification for 11β-HSD1 and GAPDH in liver. *P < 0.05 Sham vs. VSG by two-sided unpaired Student’s t–test **(J)** Western Blot of adipose tissue lysate from sham and VSG mice for 11β-HSD1 (32kDa) and GAPDH (34kDa) **(K)** Average intensity measurement from Western Blot immunoblotting quantification for 11β-HSD1 and GAPDH in adipose tissue. *P < 0.05 Sham vs. VSG by two-sided unpaired Student’s t–test **(L)** Quantitative PCR levels of *Pck1* gene expression in liver *P < 0.05 Sham vs. VSG by two-sided unpaired Student’s t–test **(M)** Quantitative PCR levels of *G6pc* gene expression in liver *P < 0.01 Sham vs. VSG by two-sided unpaired Student’s t–test. **(N)** Quantitative PCR levels of *Nr3c1 (GR)* gene expression in liver **P < 0.01 Sham vs. VSG by two-sided unpaired Student’s t–test. Data are expressed as means ± SEM.

Lean VSG-treated mice experienced no significant weight loss ten weeks post-surgery, when compared to sham operated mice (26.9 ± 2 vs. 28 ± 0.9 g) (**Figure 1A**), yet still displayed improved glucose tolerance and insulin sensitivity (**Figures 1B–E**), in line with previous findings (11). Specifically, VSG significantly enhanced glucose clearance following an intraperitoneal glucose injection (3g/kg) with an observed peak at 15 min. and glucose levels dropping to almost baseline concentration within 60 min (**Figures 1B, C**). In contrast, in sham-operated mice, glucose peaked at 30 min. and did not fully recover within the first 90min of measurement. Although all lean mice were metabolically healthy at baseline, VSG-treated mice showed enhanced insulin sensitivity, as measured by circulating glucose concentration in response to an intraperitoneal insulin injection (**Figure 1D**).

In order to assess the effect of VSG on glucocorticoid secretion, we measured corticosterone in blood collected from both VSG and sham lean mice. As previously reported in rodents, lean mice displayed low corticosterone levels during the morning (AM) and high levels in the evening (PM), while no significant differences were observed between sham and VSG-treated mice (**Figure 1E**).

### 11β-HSD1 (Hsd11b1) is downregulated in liver and adipose tissue following vertical sleeve gastrectomy

The metabolism of glucocorticoids on target tissues such as liver and adipose tissue are dependent on the enzyme 11β-HSD1. We therefore attempted to measure the expression of 11β-HSD1 in the liver and subcutaneous adipose tissue using biopsies from lean mice ten weeks post-operatively. In both tissues, *Hsd11b1* mRNA levels were significantly reduced (**Figures 1F, G**). This reduction was also confirmed at the level of 11β-HSD1 protein (**Figures 1H–K**).

### Gluconeogenesis genes are inhibited following vertical sleeve gastrectomy

Phosphoenolpyruvate carboxykinase 1 (PCK1) and glucose-6-phosphatase (G6Pase) are key enzymes in gluconeogenesis. As both are activated through the glucocorticoid receptor (GR) in the liver, we investigated their gene expression levels in VSG and sham mice (**Figures 1L, M**). Expression of both genes was significantly lowered in the liver following surgery in lean mice. Of note, GR expression was also significantly lowered after VSG (**Figure 1N**). Expression of the insulin-regulated gene glucokinase (*Gck*) (32) was also measured in the liver (**Supplementary Figure 1A**), and was not significantly altered after surgery in lean mice.

### Vertical sleeve gastrectomy restores physiological corticosterone production in HFD mice

**Figure 2:**
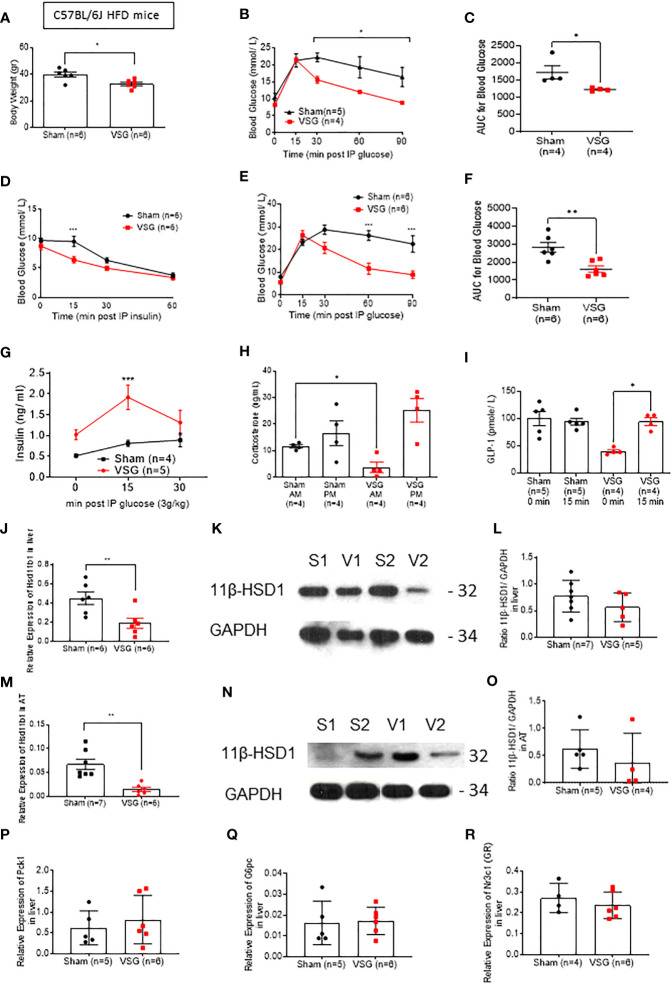
VSG restores physiological corticosterone production in HFD mice **(A)** Body weight at ten weeks following VSG (n = 6) or sham surgery (n = 6) in HFD animals **(B)** IPGTT in HFD mice (1 g/kg) 8hr fasting ten weeks after surgery *P< 0.05 Sham vs. VSG following 2-way ANOVA **(C)** Area under the Curve of **(B)**. *P < 0.05 Sham vs. VSG by two-sided unpaired Student’s t–test **(D)** Insulin tolerance test in HFD mice (1.5 IU/kg) 6hr fasting, ten weeks after surgery ***P < 0.001 Sham vs. VSG following 2-way ANOVA **(E)** IPGTT in HFD mice (3 g/kg) overnight fasting ten weeks after surgery ***P < 0.01 Sham vs. VSG following 2-way ANOVA **(F)** Area under the Curve of **(B)**. **P < 0.001 Sham vs. VSG by two-sided unpaired Student’s t–test **(G)** Insulin secretion following IPGTT (3 g/kg) and overnight fasting **(H)** Corticosterone measurement following blood collection at 0800 and 1900 within the same day, ten weeks after surgery *P < 0.05 Sham vs. VSG by two-sided unpaired Student’s t–test. **(I)** GLP-1 secretion following 3g/kg oral glucose gavage after overnight fasting *P < 0.05 Sham vs. VSG by two-sided unpaired Student’s t–test **(J)** Quantitative PCR levels of *Hsd11b1* gene expression in liver *P < 0.05 Sham vs. VSG by two-sided unpaired Student’s t–test **P < 0.01 Sham vs. VSG by two-sided unpaired Student’s t–test **(K)** Western Blot of liver lysate from sham and VSG mice for 11β-HSD1 (32kDa) and GAPDH (34kDa) **(L)** Average intensity measurement from Western Blot immunoblotting quantification for 11β-HSD1 and GAPDH in liver. **(M)** Quantitative PCR levels of *Hsd11b1* gene expression in subcutaneous adipose tissue ***P < 0.001 Sham vs. VSG by two-sided unpaired Student’s t–test **(N)** Western Blot of adipose tissue lysate from sham and VSG mice for 11β-HSD1 (32kDa) and GAPDH (34kDa) **(O)** Average intensity measurement from Western Blot immunoblotting quantification for 11β-HSD1 and GAPDH in adipose tissue. **(P)** Quantitative PCR levels of *Pck1* gene expression in liver **(Q)** Quantitative PCR levels of *G6pc* gene expression in liver **(R)** Quantitative PCR levels of *Nr3c1 (GR)* gene expression in liver. Data are expressed as means ± SEM.

Similar findings to those in lean mice were also made in mice maintained on a high fat diet (HFD) for 12 weeks (**Figures 2A–O**), although in this case surgery also affected body weight (**Figure 2A**), and insulin tolerance was significantly improved after surgery (**Figure 2D**). Glucose tolerance was also measured at 6hr fasted mice, following a 1g/kg IPGTT, in order to assess the effect of VSG at lower glucose challenges (**Figures 2B, C**). Insulin concentration was measured following an IPGTT (3g/kg) and VSG mice shown significantly higher circulating insulin compared to sham mice (**Figure 2G**). GLP-1 was also measured in overnight fasted and 15 min post 3g/kg oral glucose gavage state. Although sham-operated HFD mice displayed no difference in glucose-stimulated GLP-1 secretion before and following an oral glucose challenge, VSG appeared to restore GLP-1 circulation (**Figure 2I**), confirming our previous reports (12). Interestingly, in HFD sham mice, corticosterone concentration remained at low levels both morning (AM) and evening (PM), suggesting that the circadian rhythm of adrenal corticosterone is dysregulated in the obese state (**Figure 2H**). In contrast, in VSG-treated mice, PM corticosterone levels were restored to physiological levels, as observed in lean mice (**Figure 1F**). As with lean mice, *Hsd11b1* mRNA levels were significantly reduced (**Figures 2J–O**) and this reduction was confirmed at the level of 11β-HSD1 protein. Gluconeogenesis genes and GR were not affected in the liver of HFD mice (**Figures 2P–R**). However, expression of *Gck* was significantly lower in the VSG group (**Supplementary Figure 1B**). Since *Gck* expression is strongly induced by insulin, but not glucose, in hepatocytes (32) this result is suggestive of improved insulin sensitivity and lowered time-averaged insulin levels after surgery.

### Incretin treatment does not lower Hsd11b1 expression

**Figure 3:**
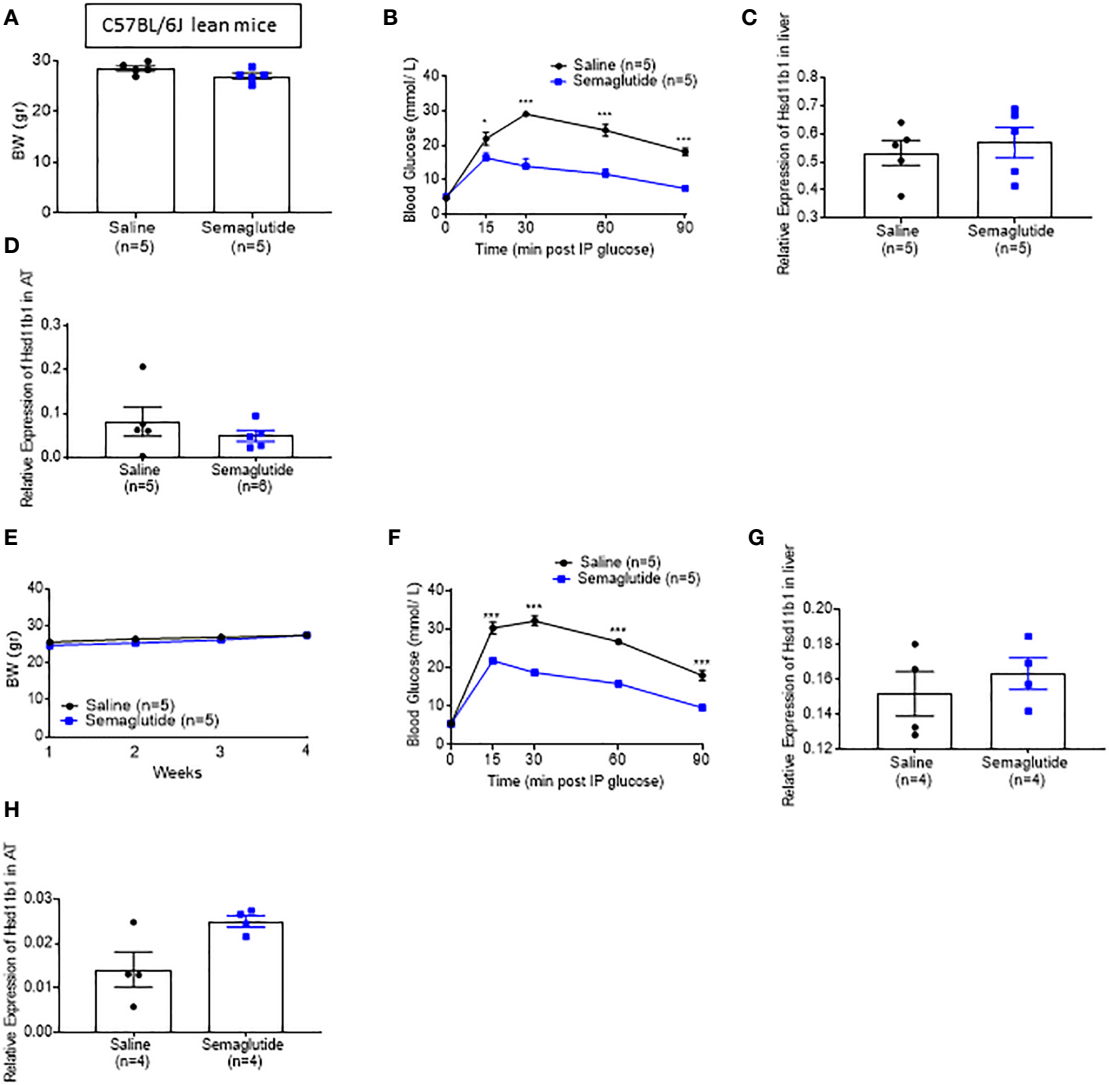
Semaglutide-induced glycaemia lowering does not decrease 11β-HSD1 levels significantly **(A)** Body weight measurement of lean mice that were treated with subcutaneous injection of 5nmol/kg Semaglutide or saline for 7 days **(B)** IP glucose tolerance test in lean mice following 7 days of Semaglutide or saline injections (3 g/kg) overnight fasting *P < 0.05, ***P < 0.001 Sema vs. saline following 2-way ANOVA **(C)** Quantitative PCR levels of *Hsd11b1* gene expression in the liver following 7 days of Semaglutide or saline treated mice. **(D)** Quantitative PCR levels of *Hsd11b1* gene expression in the subcutaneous following 7 days of Semaglutide or saline treated mice **(E)** Body weight measurement of lean mice that were treated with subcutaneous injection of 5nmol/kg Semaglutide or saline for 30 days **(F)** IP glucose tolerance test in lean mice following 30 days of Semaglutide or saline injections (3 g/kg) overnight fasting ***P < 0.001 Sema vs. saline following 2-way ANOVA **(G)** Quantitative PCR levels of *Hsd11b1* gene expression in the liver following 30 days of Semaglutide or saline treated mice. **(H)** Quantitative PCR levels of *Hsd11b1* gene expression in the subcutaneous adipose tissue following 30 days of Semaglutide or saline treated mice. Data are expressed as means ± SEM.

We next explored whether GLP-1, circulating levels [which are known to increase post bariatric surgery (33)], elevated insulin concentration or if lowered glycaemia, could play a role regulating the expression of *Hsd11b1*. To this end, we injected the GLP-1 receptor agonist (GLP-1RA) Semaglutide subcutaneously at 5nmol/kg in lean mice daily, for seven days (acute treatment) and chronically for 30 days (chronic treatment). The low dose was selected in order to prevent any loss of appetite and consequent body weight reduction. Glucose tolerance tests were performed 2 hours following a semaglutide injection. Although the body weight of the Semaglutide-treated mice did not change when compared to mice receiving saline (**Figures 3A, E**), fed glycemia was significantly lower on days 7 and 30 in the incretin vs saline-injected mice (**Figures 3B, F**). However, the expression of *Hsd11b1* in liver (**Figures 3C, G**) and subcutaneous adipose tissue biopsies (**Figures 3C, D, G, H**), obtained on the last day of treatment, was not different between Semaglutide and saline-treated groups, indicating that low glycemia or higher circulating GLP-1 does not replicate the effect observed following VSG (**Figures 1G, H**). As no difference was observed in gene expression level, protein and corticosterone levels were not investigated.

### Hsd11b1null mice display improved glycemia

**Figure 4:**
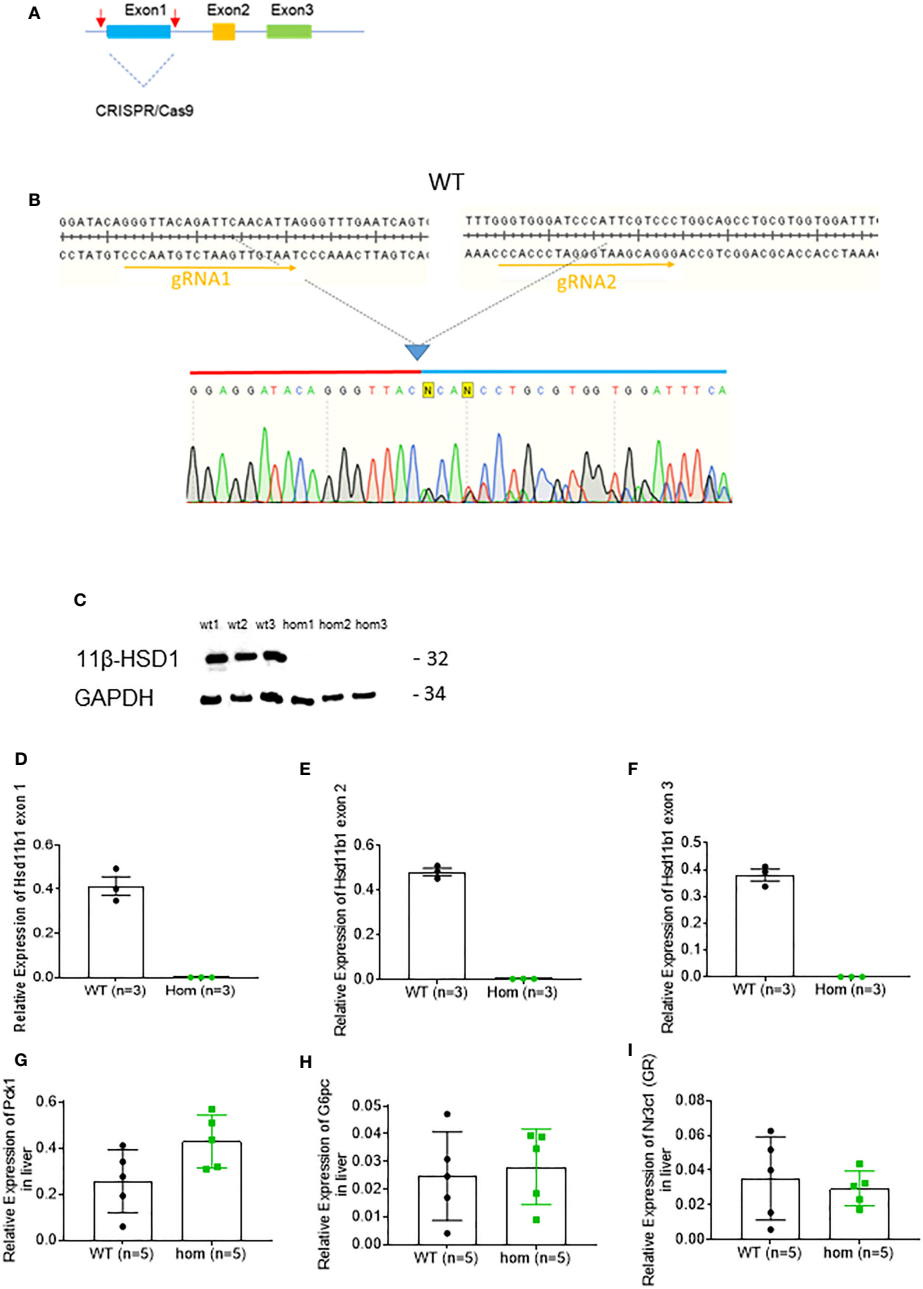
Generation and characterisation of *Hsd11b1* ^-/-^ mice. **(A)** Diagram of gene deletion *via* CRISPR/Cas9 genome editing technology. Two gRNAs flanking exon1 of *Hsd11b1* gene were designed to delete entire exon1. **(B)** Confirmation of CRISPR/Cas9 mediated genomic DNA deletion by Sanger sequencing. **(C)** Western Blot analysis of kidney cortex lysate from *Hsd11b1* ^-/-^ (hom), and Wild-type (WT) mice for 11β-HSD1^-/-^ (32kDa) and GAPDH (34kDa). **(D)** Quantitative PCR levels of *Hsd11b1* exon 1 gene expression in the liver of *Hsd11b1* ^-/-^ (hom) and WT mice. **(E)** Quantitative PCR levels of *Hsd11b1* exon 2 gene expression in the liver of *Hsd11b1* ^-/-^ (hom) and WT mice. **(F)** Quantitative PCR levels of *Hsd11b1* exon 3 gene expression in the liver of *Hsd11b1* ^-/-^ (hom) and WT mice. **(G)** Quantitative PCR levels of *Pck1* gene expression in liver. **(H)** Quantitative PCR levels of *G6pc* gene expression in liver. **(I)** Quantitative PCR levels of *Nr3c1 (GR)* gene expression in liver. Data are expressed as means ± SEM.

In order to assess the role played by lowered 11β-HSD1 levels in the effects of VSG, we generated a whole body *Hsd11b1* knockout mouse model using CRISPR/Cas9 (Methods; **Figures 4A, B**). The strategy was designed to remove the whole of exon 1 (Figure A). Deletion of exon 1 was confirmed by genomic PCR and subsequent Sanger sequencing (**Figure 4B**). Loss of 11β-HSD1 protein was confirmed by Western (immuno-) blotting (**Figure 4C**). The loss of mRNA encoded by exons 1, 2 and 3 was confirmed *via* RT-qPCR (**Figures 4D–F**, **Supplementary Figure 1**). Nevertheless, gluconeogenesis genes and GR expression were not reduced in *Hsd11b1* ^-/-^ mice, when compared to WT lean mice (**Figures 4G–I**).

**Figure 5:**
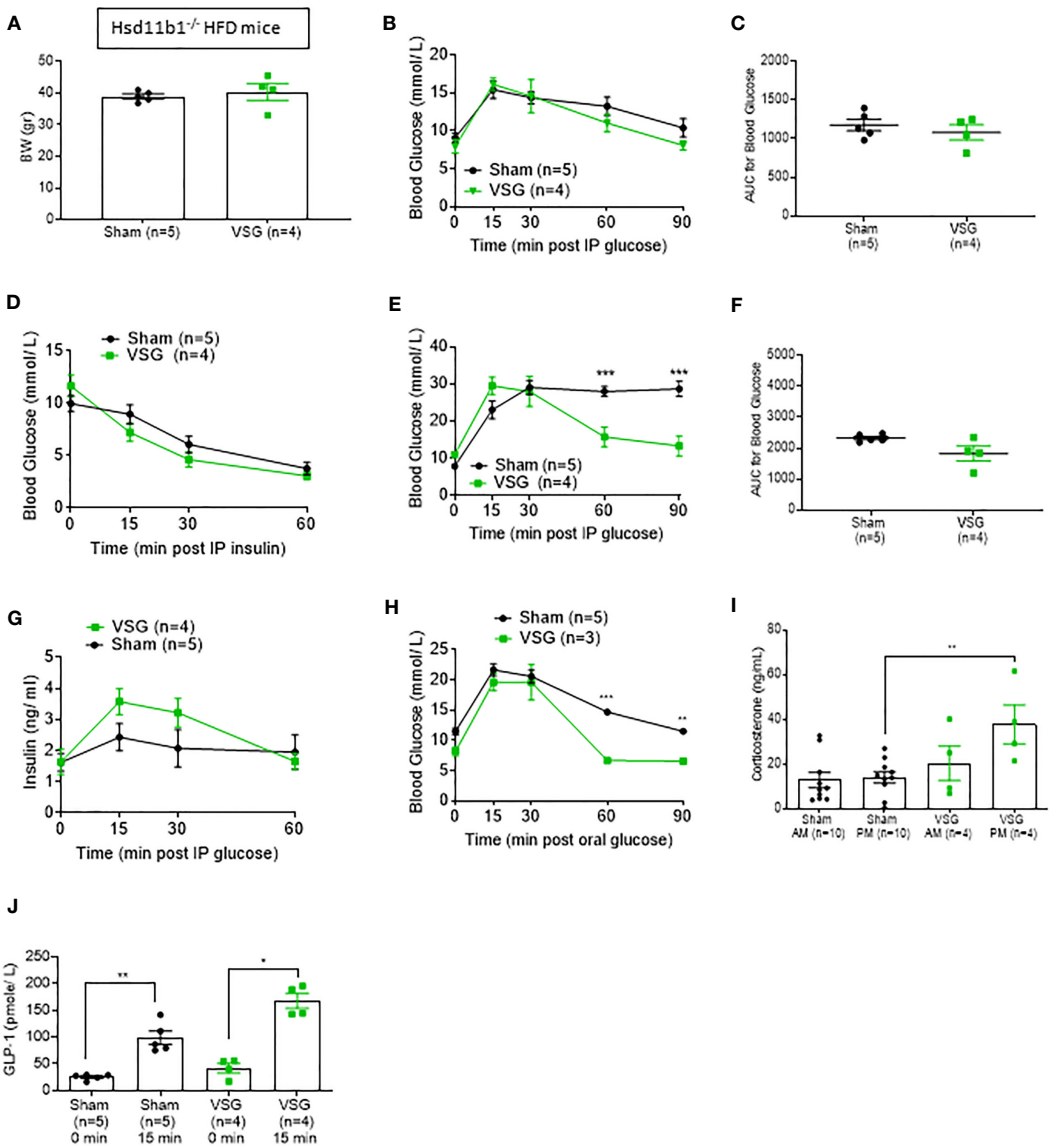
*Hsd11b1* ^-/-^ mice display improved glucose tolerance. **(A)** Body weight of *Hsd11b1* ^-/-^ HFD mice during 10 weeks post VSG and sham. **(B)** IPGTT (1 g/kg) 8hr fasting ten weeks after surgery on *Hsd11b1* ^-/-^ HFD mice **(C)** Area Under the Curve for **(B) (D)** Insulin tolerance test in HFD mice (1.5 IU/kg) 6hr fasting, ten weeks after surgery **(E)** IPGTT in HFD mice (3 g/kg) overnight fasting ten weeks after surgery, ***p < 0.001 **(F)** Area Under the Curve for **(E) (G)** Insulin concentration measured during ipGTT (3 g/kg) overnight fasting [shown in **(E)**] **(H)** Oral GTT (3 g/kg) overnight fasting 6 weeks post-op *P < 0.05 ***P < 0.001 KO sham vs. KO VSG following 2-way ANOVA **(I)** Corticosterone measurement following blood collection at 0800 and 1900 within the same day, ten weeks after surgery **P < 0.001 by two-sided paired Student’s t–test. Data are expressed as means ± SEM. **(J)** GLP-1 plasma levels following OGTT (3g/kg) overnight fasting *p < 0.05, **P < 0.001 by two-sided paired Student’s t-test.

*Hsd11b1^-/-^* mice were placed on a HFD for 12 weeks and then separated into sham and VSG groups. *Hsd11b1^+/-^* and *Hsd11b1^-/-^* mice were characterized alongside *Hsd11b1^+/+^* littermates (**Supplementary Figure 2**). Both groups displayed very similar initial weight (**Figure 5A**). Ten weeks after surgery, VSG and sham-operated *Hsd11b1^-/-^* mice displayed identical time courses of blood glucose change following an IPGTT (8h fasting, 1g/kg). Importantly, the two *Hsd11b1^-/-^* groups did not differ, but also displayed a blunted 15 min glucose peak response (**Figure 5B**). This indicates that after a low glucose challenge (1g/kg) the lack of 11β-HSD1 may contribute to the improved post-operative glucose tolerance. Comparison between *Hsd11b1^-/-^* (**Figure 5C**) and WT mice (**Figure 2C**) area under the curve (AUC) showed similar values between both sham and VSG operated *Hsd11b1^-/-^* mice (Sham 1174 ± 82, 1079 ± 92) and VSG operated WT mice (1227 ± 40), while sham operated WT mice were significantly higher (1730 ± 168). Insulin tolerance was very similar between VSG and sham-operated *Hsd11b1^-/-^* mice (**Figure 5D**).

### The effects of VSG on glucose homeostasis are attenuated in Hsd11b1null mice

Although *Hsd11b1^-/-^* mice exhibited improved glycemia when compared to WT mice during a 1g/kg glucose challenge, a higher glucose challenge (IPGTT 16h fasting, 3g/kg) showed a clear difference between VSG- operated *Hsd11b1^-/-^* and sham-operated *Hsd11b1^-/-^* mice (**Figure 5E**). This results suggests that VSG exerts a further impact on glucose homeostasis under conditions where a high glucose load presumably saturates the capacity of 11β-HSD1 inhibition. However, comparison of areas AUC for glucose excursion between VSG- operated *Hsd11b1^-/-^* and sham-operated *Hsd11b1^-/-^* mice revealed no significant difference (**Figure 5F**). Similar to the 1g/kg glucose challenge (Fig 2C, 5C), comparison of IPGTT 3g/kg AUC between sham-operated WT mice (2827± 268.5) (**Figure 2F**) and sham-operated *Hsd11b1^-/-^* mice (2299± 149) (**Figure 5F**) showed a significant reduction of glucose in *Hsd11b1^-/-^* mice.

Further evidence of the impact of *Hsd11b1* deletion on the outcomes of VSG was provided by measurement of increased insulin concentration in response to a 3g/kg IPGTT (**Figure 5G**), when compared to WT mice. VSG-operated WT mice had higher insulin concentrations when compared to WT sham (**Figure 2G**). However, VSG-operated *Hsd11b1^-/-^* mice did not display significantly higher insulin levels when compared to *Hsd11b1^-/-^* sham-operated mice. Both VSG and sham- operated *Hsd11b1^-/-^* groups showed over 2-fold higher insulin concentration when compared to VSG-operated WT mice. An oral GTT (3g/kg 16hr fasting) confirmed the additional beneficial effect of VSG over sham surgery (**Figure 5H**). Of note, *Hsd11b1^-/-^* VSG-treated mice also demonstrated an improved corticosterone secretion pattern (low levels in the morning AM – higher levels in the evening PM), when compared to *Hsd11b1^-/-^* sham mice (**Figure 5I**), similar to HFD WT mice (**Figure 2H**).

### 11β-HSD1 deletion restores GLP-1 secretion in HFD mice

Circulating GLP-1 levels were measured after OGTT in *Hsd11b1^-/-^* mice (**Figure 5J**). Both VSG and sham- operated *Hsd11b1^-/-^* mice showed a significant increase of GLP-1 15 min. following a glucose oral gavage (**Figure 5H**). Although VSG-operated WT mice were previously confirmed to restore GLP-1 secretion (**Figure 2I**), this effect is now also shown in *Hsd11b1^-/-^* sham-operated mice, indicating that the euglycemic effect displayed in the absence of 11β-HSD1 could be through an improvement in incretin secretion.

## Discussion

In this study we explored, for the first time to the best of our knowledge, the possible existence of a physiologically and pathophysiologically relevant gut-glucocorticoid axis. We utilised a gastrointestinal procedure that excludes most of the stomach and investigated the effects this would have on both circulating cortisol secretion and tissue-specific cortisol metabolism, while removing the confounding effects of weight loss. Furthermore, we investigated potential mediators of this axis by replicating the post-operative low glycemia pharmacologically by using Semaglutide. Finally, we attempted to replicate the effect of the surgery on tissular glucocorticoid regulation by knocking out the gene encoding the cortisol-activating enzyme 11β-HSD1.

Currently, the two most widely used types of bariatric surgery are VSG and Roux-n-Y-Gastric-Bypass (34). Despite the similar metabolic outcomes between the two procedures, VSG is reported to have lower mortality in mice (35, 36), but also allows for gradual weight regain, which is why it was chosen for this study (8, 37). Both lean and HFD-induced hyperglycemic models were studied in order to compare glucocorticoid responses to VSG in models of health and disease. In the lean model, no weight loss was observed post-operatively, yet glucose clearance increased significantly. In the HFD model, mild weight loss was observed at week ten post-operatively, yet glucose clearance rate increased significantly, as we have previously reported (12). These findings validate the metabolic phenotype of the VSG mouse model as reported in multiple studies (35, 38). Of note, insulin sensitivity curves between lean and HFD mice were almost identical (39) demonstrating potentially similar mechanisms of improvement. We have also previously reported a significant increase of insulin concentration following VSG in lean and HFD mice (11, 12).

As an initial assessment of the glucocorticoid regulation following VSG, we measured circulating corticosterone in the early morning and late afternoon in all mice. This was done in ten weeks after surgery to avoid measuring increased post-operative stress-related corticosterone levels. In healthy mice, physiological corticosterone levels are low in the morning and elevated in the evening (40), and both VSG and sham-treated lean mice demonstrated this pattern. Interestingly, HFD sham-treated mice showed no increase in corticosterone during the day, indicating impaired regulation of adrenal secretion. However, VSG restored normal corticosterone levels in HFD mice, matching the concentration found in lean mice, with a physiological increase in circulating glucocorticoid concentration apparent in the evening. This is of importance as glucocorticoids, within the normal physiological range, are vital for metabolic, inflammatory and cardiovascular processes and an evening increase (equivalent of a morning increase in humans) could be associated with an overall improvement in lipid metabolism, immune response and vascular health (41). Moreover, the increase in corticosterone may contribute to improving functional enterocyte morphology and proliferation, and therefore gastrointestinal metabolic health in the long term (42).

Despite the reported link between glucocorticoids and obesity (20), it is still unclear how circulating glucocorticoid levels are modulated following weight loss, and the cause of weight loss seems to be a confounder (43). Previous reports have reported dysregulated cortisol levels in obesity, that remain unchanged, decreased, or increased following diet-induced weight loss, probably as a result of the stress associated with dieting, or the type of nutrients included in a diet (44–46). Several studies have attempted to study the regulation of cortisol levels following bariatric surgery in clinical settings (47–49). However, differences in the methodology and patient inclusion criteria (type of surgery, post-operative time, body mass index (BMI) or presence of T2D and/or eating disorders), have resulted in conflicting results. Our findings are supported by those of Valentine et al. who found a 54% rise in morning saliva cortisol levels at six and twelve months after VSG in women with obesity, but no differences in night-time samples, which is in line with our findings (50).

Although the concentration of circulating glucocorticoids is important for their action, the effects of glucocorticoids on target tissues such as liver and adipose tissue are dependent on 11β-HSD1 activity (18). Previous studies have shown that mice overexpressing 11β-HSD1 in adipose tissue develop visceral obesity, insulin resistance and dyslipidemia (51), while liver-specific 11β-HSD1 overexpression results in insulin resistance and hypertension, but not obesity (52). In our study, we found that 11β-HSD1 expression is significantly inhibited in the liver and adipose tissue in both lean and HFD VSG-treated mice when compared to sham groups, pointing towards reduced tissular glucocorticoid activity which can potentially be a contributing factor to the significant increase in insulin sensitivity observed. Expression of 11β-HSD1 was previously shown to be higher in patients with obesity, when compared to lean individuals (53). We observed this finding in our study, as liver 11β-HSD1/*Hsd11b1* expression levels in obese mice were 5-fold higher than lean mice. Though this could mean that 11β-HSD1/*Hsd11b1* expression reduction is due to post-operative weight loss, VSG-treated lean animals also demonstrated significant expression inhibition. This indicates that 11β-HSD1/*Hsd11b1* expression is regulated by more than adiposity.

In the liver, glucocorticoids have been shown to stimulate gluconeogenesis through the glucocorticoid receptor (54). Kalvisa et al. (55) have previously shown that insulin signaling and reduced glucocorticoid receptor activity decrease postprandial gene expression in liver. Specifically, they suggested that postprandial suppression of circulating corticosterone and increased insulin levels independently regulate the mRNA levels of a subset of feeding-regulated genes, such as *Pck1* and *G6pc*, in the liver. In our study, *Pck1* and *G6pc* gene expression is inhibited in lean mice, possibly followed by the inhibition of the glucocorticoid receptor. Though it would be tempting to link the reduced 11β-HSD1 levels as the background mechanism of reduced gluconeogenesis genes, VSG-treated HFD mice (that demonstrated the same low 11β-HSD1 levels as lean mice) showed no difference in *Pck1* and *G6pc* gene expression when compared to WT sham mice. Moreover, lean *Hsd11b1^-/-^* mice also demonstrated no difference in glucocorticoid receptor, in *Pck1* and *G6pc* gene expression when compared to WT littermate mice, as previously reported in whole body (56) and liver-specific (57) knockout models. This raises the question of whether the glucocorticoid receptor is the driving background mechanism for some of the euglycemic hepatic effects we observed in the lean mice, and how is it altered after VSG.

In an effort to elucidate the mechanism(s) that links VSG, a gastric procedure, to adrenal function, we explored the role of GLP-1, a post-prandially released hormone that enhances insulin secretion and lowers glucose concentration (58). This incretin hormone has also been shown to increase significantly following VSG, and to regulate glucocorticoid secretion (16, 59). We acutely and chronically injected a low dose of the GLP-1RA Semaglutide, to avoid significant body weight loss while achieving significantly lower glycemia, in lean mice as previously reported (11). *Hsd11b1*/11β-HSD1 expression was not affected in either the liver or in the adipose tissue of Semaglutide-treated mice, suggesting that the observed effects are not driven by the reduction in blood glucose, insulin or a direct action of GLP-1.

In order to understand the role of 11β-HSD1 in the observed effects of VSG, we developed an 11β-HSD1 deficient mouse line (*Hsd11b1^-/-^*) by deleting exon 1 of the *Hsd11b1* gene. The importance of this experiment lay primarily with the sham group, as the aim was to determine to what extent the knock out of a single gene can replicate the effects of surgery. Following 12 weeks on HFD and a VSG or sham procedure, both groups of *Hsd11b1^-/-^* mice displayed no difference in post-operative body weight. Though this may initially suggest that VSG was unable to cause weight loss in *Hsd11b1^-/-^* mice, comparison with body weights in WT miuce that had undergone sham surgery shows that it is sham-operated *Hsd11b1^-/-^* that do not gain weight. This is in agreement with previous studies showing *Hsd11b1^-/-^* mice are protected from the accumulation of visceral fat following high-fat diet-feeding (60), and also demonstrate reduced food intake (61). The improved metabolic phenotype of the knockout sham mice is further supported by their behavior during a 1g/kg glucose tolerance test, where glucose concentrations of sham-treated *Hsd11b1^-/-^* mice were identical so VSG-treated *Hsd11b1^-/-^* mice and VSG-treated WT mice, and significantly lower than sham-treated WT mice. However, at higher glucose challenge (3g/kg) sham-treated *Hsd11b1^-/-^* mice behavior bears more resemblance to WT sham mice, indicating a saturation of the positive genotypic effect. This observation suggested that *Hsd11b1* inhibition may contribute to bariatric surgery-associated euglycemia, but it is not the sole mechanism involved.

Although *Hsd11b1^-/-^* mice did not demonstrate a highly significant increase in insulin sensitivity, compared to WT mice, they did have a relatively high insulin concentration even at basal level, before any glucose challenge. Though this would normally indicate insulin resistance and high glucose levels, corresponding AUC glucose values show lower glucose concentration in *Hsd11b1^-/-^* mice compared to WT mice when challenged with the same amount of glucose (3g/kg). This finding is in contrast with the fact that *Hsd11b1* is a disallowed gene in pancreatic islet α and β cells (17), indicating that the effect may be driven in an indirect way, such as incretin stimulation. Indeed, although plasma GLP-1 secretion is dysregulated in HFD sham-operated mice, GLP-1 concentration was maintained in physiological levels in obese *Hsd11b1^-/-^* sham mice, again resembling both VSG-treated *Hsd11b1^-/-^* mice and VSG-treated WT mice, as previously shown (12). This result also indicates an effect of reduced tissular glucocorticoid activity on GLP-1 secretion improvement, possibly through an effect on L-cells, providing an additional mechanism of improved insulin sensitivity.

Even though there is evidence that bariatric surgery can affect the HPA axis (47, 62), there is more controversy around the direction of this regulation. The long-term effects of bariatric surgery on cortisol levels are also unclear. In clinical settings, hepatic 11β-HSD1 activity is primarily measured by calculating serum cortisol/cortisone ratio, but direct tissular expression requires biopsies which are usually not available. In patients with severe obesity following RYGB, 11β-HSD1 activity was increased in the liver but expression was decreased in the subcutaneous adipose tissue (24, 25, 63) highlighting that regulation of cortisol metabolism in obesity and after weight loss is highly tissue specific. In our study, we demonstrated that VSG improves insulin sensitivity and lowers 11β-HSD1 expression in both physiological and pathophysiological conditions. Moreover, VSG appears to restore physiological corticosterone levels in pathophysiological conditions. Finally, we provide evidence that genetic elimination of *Hsd11b1^-/-^* in mice may improve glucose tolerance in a manner that partly resembles the euglycemic effect of VSG.

*Limitations:* Due to the high severity of the VSG surgery, only male mice were investigated. This study should therefore be repeated in the future in female mice and the results compared with those reported here. Moreover, all wild type studies were performed as proof of concept with purchased mice rather than with littermates to the *Hsd11b1* null animals. Liver adiposity measurements would be beneficial to understand the rate of lipolysis post-operatively. As with clinical studies, chronic cortisol measurements are required to further validate our findings. Additionally, 11β-HSD1 tissular activity should be further explored in mice. Similar to the Semaglutide study, more factors must be explored to identify a potential hormonal mediator(s) between the gut and the adrenal secretion regulation. Finally, our results must extend to the clinical settings, especially in patients with Cushing’s syndrome following bariatric surgery.

## Conclusions

We show here, for the first time, that VSG maintains normal corticosterone levels but inhibits tissular glucocorticoid metabolism in lean animals, by lowering 11β-HSD1 mRNA and protein, and that this effect is likely to be weight loss and glycaemia-independent. Moreover, we have demonstrated that corticosterone circulation is restored in obese VSG animals, while tissular glucocorticoid metabolism is inhibited, even after weight regain. Insulin sensitivity and glucose clearance were enhanced in all groups. These observations demonstrate a potential new mechanism of T2DM remission after bariatric surgery.

## Data availability statement

The original contributions presented in the study are included in the article/**Supplementary Material**. Further inquiries can be directed to the corresponding author.

## Ethics statement

The animal study was reviewed and approved by All animal procedures undertaken were approved by the British Home Office under the UK Animal (Scientific Procedures) Act 1986 (Project License PA03F7F0F to I.L.) with approval from the local ethical committee (Animal Welfare and Ethics Review Board, AWERB), at the Central Biological Services (CBS) unit at the Hammersmith Campus of Imperial College London.

## Author contributions

EA designed the study, undertook the mouse studies and all data analyses. EA and GR supervised the study. MH designed gRNAs and genotyping protocol for knockout mouse line. LL-N and IL assisted with mouse studies. EA, IC, AM, and GR wrote the manuscript with contributions from all authors. All authors contributed to the article and approved the submitted version

## Funding

EA was supported by a grant from the Rosetrees Trust (M825) and from the British Society for Neuroendocrinology. IC was supported by the Society for Endocrinology (19ES001). GR is supported by a Wellcome Trust Investigator (212625/Z/18/Z) Award, MRC Programme grants (MR/R022259/1, MR/J0003042/1, MR/L020149/1), and Experimental Challenge Grant (DIVA, MR/L02036X/1), MRC (MR/N00275X/1), and Diabetes UK (BDA/11/0004210, BDA/15/0005275, BDA 16/0005485) grants and an Innovation Canada John R. Evans Leaders Award. This project has received funding from the European Union’s Horizon 2020 research and innovation programme *via* the Innovative Medicines Initiative 2 Joint Undertaking under grant agreement No. 115881 (RHAPSODY) to GR. IL was supported by a project grant from Diabetes UK (16/0005485).

## Conflict of interest

GR has received grant funds from Servier Laboratories and Sun Pharmaceutical Industries Ltd. These funders were not involved in any of the studies discussed here.

The remaining authors declare that the research was conducted in the absence of any commercial or financial relationships that could be construed as a potential conflict of interest.

## Publisher’s note

All claims expressed in this article are solely those of the authors and do not necessarily represent those of their affiliated organizations, or those of the publisher, the editors and the reviewers. Any product that may be evaluated in this article, or claim that may be made by its manufacturer, is not guaranteed or endorsed by the publisher.
