# Supplementary figures and images for: Vertical sleeve gastrectomy normalizes circulating glucocorticoid levels and lowers glucocorticoid action tissue-selectively in mice
Source: Front Endocrinol (Lausanne). 2022 Sep 29;13:1020576. doi: 10.3389/fendo.2022.1020576 (PMC9556837; doi:10.3389/fendo.2022.1020576)

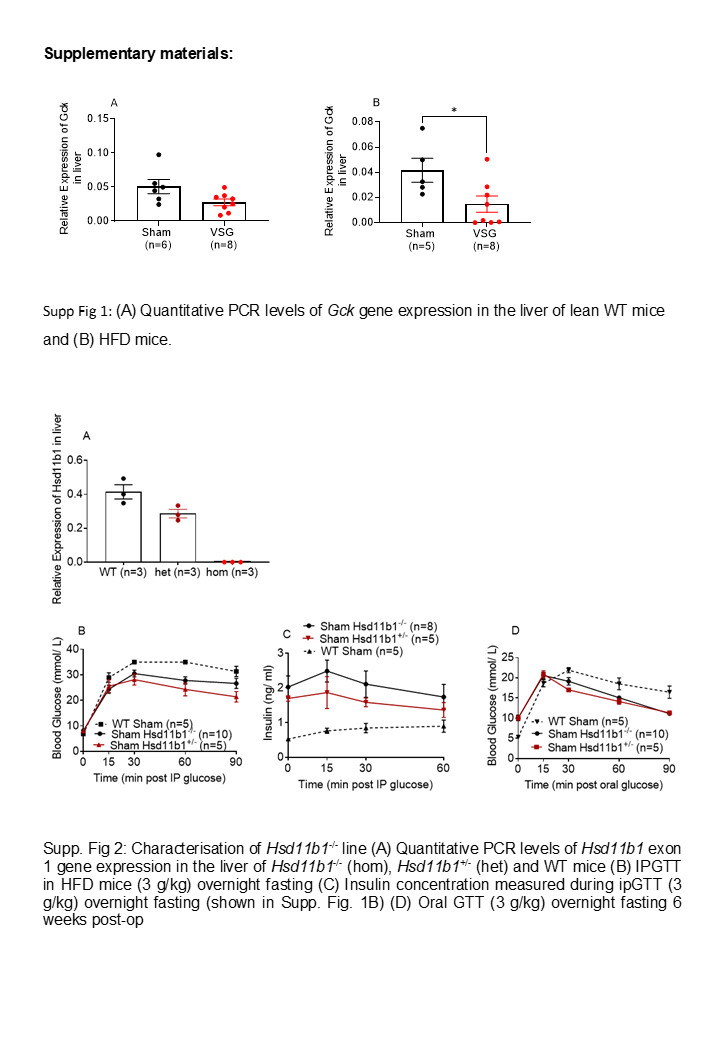

Supplement: Supplementary file 1 [file Image_1.tif]
